# Optimizing *Shigella* isolation: a multi-site evaluation of laboratory culture methods for *Shigella* detection, speciation, and serotyping with different transport media and sample types in the Enterics for Global Health study

**DOI:** 10.1128/jcm.01279-25

**Published:** 2026-02-26

**Authors:** Md Taufiqur Rahman Bhuiyan, Jie Liu, Jane Juma, Bri'Anna Horne, Aneeta Hotwani, Henry Badji, Victor Adrian Maiden, John Benjamin Ochieng, Lucero A. Romaina-Cachique, Laura Riziki Aluoch, Lilian Achieng Ambila, Fatima Aziz, Mary Charles, Erika L. Feutz, Paul F. Garcia Bardales, M. Jahangir Hossain, Junaid Iqbal, Md Taufiqul Islam, Abdoulie Jabang, Sheikh Jarju, Khuzwayo C. Jere, Furqan Kabir, Flywell Kawonga, Adama Mamby Keita, Farhana Khanam, Katia Manzanares Villanueva, Md Parvej Mosharraf, Richard Omore, Maribel Paredes Olortegui, Patricia B. Pavlinac, James A. Platts-Mills, Elizabeth T. Rogawski McQuade, Queen Saidi, Khandra T. Sears, Milagritos D. Tapia, Awa Traore, Firdausi Qadri, Pablo Penataro Yori, Farah Naz Qamar, Samba O. Sow, Karen L. Kotloff, Sharon M. Tennant, Eric R. Houpt, Jennifer Cornick, Ousman Secka, on behalf of the EFGH Consortium

**Affiliations:** 1 International Centre for Diarrhoeal Disease Research, Bangladesh 56291, Dhaka, Bangladesh; 2 School of Public Health, Qingdao University 571594, Qingdao, Shandong, China; 3 Center for Vaccine Development Mali 560812 , Bamako, Mali; 4 Center for Vaccine Development and Global Health, University of Maryland School of Medicine 12264, Baltimore, Maryland, USA; 5 Department of Pediatrics and Child Health, Aga Khan University 196391, Karachi, Pakistan; 6 Medical Research Council Unit The Gambia at the London School of Hygiene & Tropical Medicine, Banjul, The Gambia; 7 Malawi Liverpool Wellcome Trust Research Programme, Blantyre, Malawi; 8 Center for Global Health Research, Kenya Medical Research Institute, Kisumu, Kenya; 9 Asociacion Benefica Prisma, Iquitos, Loreto, Peru; 10 Department of Global Health, University of Washington 150331, Seattle, Washington, USA; 11 London School of Hygiene & Tropical Medicine 4906, London, United Kingdom; 12 Institute of Infection, Veterinary and Ecological Sciences, University of Liverpool 4591, Liverpool, United Kingdom; 13 Department of Medical Laboratory Sciences, School of Life Sciences and Health Professions, Kamuzu University of Health Sciences 37610, Blantyre, Malawi; 14 Department of Epidemiology, University of Washington, Seattle, Washington, USA; 15 Division of Infectious Diseases and International Health, University of Virginia 2358, Charlottesville, Virginia, USA; 16 Department of Epidemiology, Emory University 209733, Atlanta, Georgia, USA; 17 Department of Natural and Physical Sciences, Baltimore City Community College 32723, Baltimore, Maryland, USA; 18 Department of Medicine, University of Maryland School of Medicine 12264, Baltimore, Maryland, USA; Maine Medical Center Department of Medicine, Portland, Maine

**Keywords:** gastrointestinal infections, global child health, pediatric infectious diseases, infectious diseases, global health

## Abstract

*Shigella* is a leading cause of diarrhea and dysentery in children younger than 5 years of age in low-resource settings, and several vaccines are in development. Due to its fastidious nature, *Shigella* can be difficult to culture, and eventual vaccine trials will need to optimize the isolation of *Shigella* to ensure efficient sample sizes. In the recently concluded Enterics for Global Health (EFGH) *Shigella* Surveillance study, we compared *Shigella* culture isolation rates between rectal swab vs. whole stool, between two swabs vs. one, and between Cary Blair (CB) vs. modified-Buffered Glycerol Saline (mBGS) transport media to identify the optimal methods for *Shigella* recovery by microbiologic culture. Among 9,476 children aged 6–35 months enrolled in the EFGH study from seven country sites, *Shigella* isolation rates did not differ significantly between CB (7.8%) and mBGS (7.9%) (*P* > 0.99). Using two swabs improved the detection rates (9.3%), compared with one swab (7.9%) (*P* < 0.001). Among the 2,048 children from Bangladesh and The Gambia, where both rectal swabs and whole stool were collected from the same children, rectal swabs were found to be non-inferior to whole stool for *Shigella* culture (12.4% and 12.7%, respectively, with a difference of −0.29% [95% CI: −0.83% to 0.24%]). To optimize *Shigella* recovery for future multi-country vaccine trials, we recommend collecting two flocked rectal swabs in CB or mBGS media with strict adherence to transit conditions—an approach proven feasible across EFGH sites.

**Clinical Trials:**

This study is registered with ClinicalTrials.gov as NCT06047821.

**IMPORTANCE:**

*Shigella* is difficult to isolate by culture, making optimized sampling and transport essential for microbiologic confirmation. As *Shigella* vaccines are tested for efficacy and eventual licensure, it is critical that the laboratory methods are optimized to avoid missing children with shigellosis. In the multi-country Enterics for Global Health (EFGH) study, which enrolled over 9,000 children with diarrhea, rectal swabs and whole stool recovered a similar number of *Shigella* isolates, with two swabs improving detection over a single swab. Cary Blair (CB) and modified-Buffered Glycerol Saline (mBGS) transport media also resulted in similar isolation rates. To maximize outcome ascertainment in future vaccine trials, two flocked rectal swabs with transport in either medium are recommended to balance sensitivity, feasibility, and scalability.

## INTRODUCTION

*Shigella* species (spp.) are a leading cause of moderate-to-severe diarrhea among children younger than 5 years of age, contributing to more than 80,000 child deaths per year (1). *Shigella* is divided into four species (*Shigella flexneri*, *Shigella sonnei*, *Shigella boydii,* and *Shigella dysenteriae*), with the majority of shigellosis caused by *S. flexneri* and *S. sonnei* (2). Although *S. flexneri* exists as multiple serotypes, of which *S. flexneri* 2a, 3a, and 6 are the most common globally, there is geographic heterogeneity in burden and serotype/subserotype distribution (2–4). Based on global burden data, it is estimated that a vaccine targeting *S. flexneri* 2a, 3a, and 6, and *S. sonnei* could prevent more than 80% of shigellosis cases globally (2). Multiple vaccine candidates are under development, and it is expected that at least one candidate will be tested in a Phase III licensure trial within the next 5 years (5).

Stool culture has long been considered the gold standard method for diagnosis of shigellosis, and it remains a prerequisite step for downstream speciation, serotyping, and antimicrobial susceptibility testing (6). However, the sensitivity of *Shigella* culture varies by disease severity and stage, with higher culture positivity in severe cases and optimal detection when stool is sampled early in illness (7, 8). Other variables that may affect stool culture sensitivity include laboratory capacity, sample type, handling conditions, transportation time, and the type of media used to preserve samples prior to plating (9).

Rectal swabs are used for *Shigella* culture when whole stool is unavailable or when a prompt sample collection is required prior to antibiotic administration. Several studies in Europe, the Americas, and Asia (9–14), which have compared rectal swabs with whole stool for diagnostic performance of enteric pathogens causing diarrhea, have consistently shown that both methods are comparable, with no significant differences (10, 12). Although data from Africa are limited, studies conducted in Rwanda and Botswana (15–17) have shown that rectal swabs can serve as a practical alternative to bulk stool, particularly in settings where stool collection may be challenging, and rapid diagnostics are essential. Use of flocked rectal swabs is an important development for enteropathogen identification when whole stool is not available (10). Notably, rectal swabs have demonstrated superior performance for molecular testing (15). However, no studies using culture methods have yet directly compared *Shigella* isolation from rectal swabs and whole stool in high-burden settings; therefore, the current study was conducted to address this gap.

Transport media are used when fecal samples cannot be cultured immediately, as the fastidious nature of *Shigella* makes it sensitive to suboptimal conditions. Cary Blair (CB) medium is the most widely used medium for fecal sampling due to its ability to preserve bacterial viability during transport (18). Its use, however, has not been studied in low- and middle-income countries (LMICs), where variations in storage conditions, particularly in rural settings with limited infrastructure, can negatively affect sample quality and, consequently, diagnostic accuracy (19). Buffered glycerol saline (BGS) is an alternative transport medium with some evidence of it yielding a higher *Shigella* culture positivity than CB, albeit in small studies (20, 21). Additionally, one study showed that a modified version of BGS (0.5% agar and reduced glycerol to 15%) showed enhanced recovery of *Shigella* (20).

As vaccine trials are planned with the goal of preventing culture-confirmed *Shigella* infections*,* optimizing detection methods from fecal samples will be critical to the success of *Shigella* vaccine development. In the seven-country Enterics for Global Health (EFGH) *Shigella* surveillance study, which enrolled children aged 6–35 months seeking care for diarrhea, we aimed to identify the optimal sample type and transport media for *Shigella* stool culture. Findings from this study will directly inform laboratory protocols for future *Shigella* vaccine licensure trials.

## MATERIALS AND METHODS

The EFGH *Shigella* surveillance study protocol is described in detail elsewhere (22, 23). In brief, diarrheal cases were enrolled from public health facilities and hospitals in seven countries: Malawi, Kenya, Mali, The Gambia, Bangladesh, Pakistan, and Peru. After verbal screening consent was obtained from caregivers, children between the ages of 6–35 months attending EFGH health facilities with diarrhea, defined as three or more loose or watery stools in the 24 h prior to enrollment, were screened during EFGH working hours, using a standardized questionnaire. Written informed consent for the study was obtained from caregivers of eligible children after screening. Other eligibility criteria were listed previously (23). Detailed clinical demographic information was collected through standardized case report forms and physical examinations.

Two nylon flocked rectal swabs (COPAN^©^, USA) were collected from enrolled children immediately after screening or as close to screening as possible, preferably prior to antibiotic administration (if prescribed). Rectal swabs were collected as described previously (22) using standardized culture approaches across all participating sites to ensure consistency and reliability in *Shigella* detection. Briefly, at enrollment, two rectal swabs were collected from each child. Children were positioned either on their side or stomach, typically on the caregiver’s lap, and rectal swabs were gently inserted into the child’s rectum, fully rotated three times, and slowly removed. One rectal swab was then immediately placed in CB medium, and the other in mBGS. All study personnel were trained using a unified protocol, standard operating procedures (SOPs) were implemented, and quality assurance measures, including observation by study monitors, were established to monitor adherence and minimize variability across sites.

The rectal swab order for each respective media was swapped halfway through the study to avoid concern that the order of swabbing influenced the quantity of fecal sample available to culture. Whole stool was also collected from children in two of the seven EFGH country sites (Bangladesh and The Gambia) if produced within 6 h of screening and prior to the child being ready to return home. To ensure whole stool was prepared similarly to rectal swabs for the swab/stool comparison, a nylon-flocked rectal swab was used to touch the stool targeting bloody, slimy, mucoid, or watery areas and placed into the appropriate tubes with CB transport media (first half of the study period) or mBGS media (second half of the study period).

Immediately after collection, all fecal samples (rectal swabs and whole stool) were placed in a cold box and maintained at 2°C–8°C, monitored for temperature excursions using a single-use 2°C–8°C temperature monitor (WarmMark, Spotsee company or 3M). Samples were transferred to an in-country central laboratory within 16 h of collection. Upon reception, an accessioning form was completed, and samples eligible for processing were cultured for *Shigella* isolation. The microbiological culture process has been described in detail previously (23). In brief, swabs in mBGS and CB were streaked on MacConkey (MAC) and Xylose Deoxycholate (XLD) agar and incubated for 18–24 h at 35°C–37°C. Up to 10 suspected *Shigella* non-lactose fermenter (NLF) colonies were subcultured on trypticase soy agar (TSA) and screened using a series of biochemical tests. Pure NLF colonies from the TSA were subcultured on triple sugar iron (TSI), motility indole ornithine (MIO) or motility indole urea (MIU), urea, lysine decarboxylase, and TSA and incubated as described previously (23). Pure, fresh suspected *Shigella* colonies on TSA were identified as *Shigella* and serotyped using latex agglutination. Latex agglutination is a rapid diagnostic method that utilizes latex beads coated with specific antibodies (antisera) to detect bacterial antigens on the cell surface. When the target antigen is present and well-isolated, the antibody-coated beads bind to it, resulting in visible clumping. Polyvalent antisera were used to identify *S. dysenteriae*, *S. flexneri*, *S. boydii*, and *S. sonnei*. If a colony was identified as *S. flexneri*, monovalent antisera were used to identify the *S. flexneri* serotype as previously described (23).

To harmonize methods across different sites, a joint working group was convened comprised of representatives from coordinating and implementing teams. The group developed a set of consensus standard operating procedures (SOPs), worksheets, and data collection forms for every component of the laboratory methodology, including sample collection and transport, media preparation, and steps for primary *Shigella* isolation, biochemical identification, serotyping with agglutination assays, and susceptibility testing (23). Essential laboratory supplies were procured centrally as needed. The team followed quality control (QC) and quality assurance procedures, along with site visits approximately every 6 months to review the implementation and adherence to protocols.

The protocol for this study was approved by the respective institutional review boards (IRBs) for each country study site and their affiliates. This study was conducted according to Good Clinical Practice (GCP), including Good Clinical Laboratory Practice (GCLP), the Declaration of Helsinki, IRB, and local rules and regulations specific to each EFGH country.

### Statistical analysis

The percentage of rectal swab samples from each media that isolated *Shigella* were summarized by serotype and country site, and two-sided 95% confidence intervals were calculated assuming a binomial distribution. McNemar’s test of superiority was employed to assess whether either medium had significantly different *Shigella* isolation proportions based on *P* < 0.05. To reduce the familywise error rate from multiple comparisons, Holm-Bonferroni adjustments were applied to *P*-values, considering all comparisons within serotypes as a family.

The percentage of whole stools and rectal swabs that isolated *Shigella* was calculated both overall and at each country site and further stratified by *Shigella* species. Because two swabs were collected for each participant, we considered only the *Shigella* isolation from the first rectal swab collected from each child, which matches the media type that the same child’s whole stool was transported in. Separately, a sensitivity analysis was performed to compare *Shigella* isolation from either of two rectal swabs with whole stool. To determine whether rectal swabs were inferior to whole stool for isolating *Shigella,* McNemar’s test of non-inferiority was used. We computed a one-sided 95% CI for the absolute difference in proportions between sample types. The lower bound of the CI was compared with the non-inferiority margin of an absolute difference of 0.01, or 1%, to establish non-inferiority.

The data used in this analysis were generated for the EFGH study and are available on the Vivli data sharing platform (Vivli DOI: PR00011860).

## RESULTS

The demographic characteristics of the 9,476 children aged 6–35 months enrolled in the EFGH study are listed in Table 1. The majority of children fell in the 12–23 months age range (46.1%) or the 6–11 months age range (36.7%). The proportion of enrolled cases presenting with dysentery ranged from 6.6% in Mali to 17.2% in The Gambia. Dehydration and hospitalization also varied substantially between countries. Swabs were collected from all enrolled children in EFGH and whole stool from 1,097 (80.6%) children in Bangladesh and 975 (69.7%) children in The Gambia. Fourteen whole stool samples were excluded in the analyses due to having been collected more than 6 h from rectal swab collection. Rectal swab samples were placed in transport media on average 0–2 min after collection, and the average time between collection and arrival at the central laboratory for primary plating ranged from 1.4 h in The Gambia to 4.4 h in Malawi.

**TABLE 1:** Participant characteristics and sample collection. n (%) or median [IQR].

	Bangladesh	Kenya	Malawi	Mali	Pakistan	Peru	The Gambia	Overall
Enrolled	1,361	1,400	1,399	1,400	1,400	1,117	1,399	9,476
**Participant demographics**								
Age (months)								
6-11	572 (42.0)	560 (40.0)	478 (34.2)	600 (42.9)	454 (32.4)	365 (32.7)	446 (31.9)	3,475 (36.7)
12-23	588 (43.2)	595 (42.5)	627 (44.8)	613 (43.8)	646 (46.1)	585 (52.4)	718 (51.3)	4,372 (46.1)
24-35	201 (14.8)	245 (17.5)	294 (21.0)	187 (13.4)	300 (21.4)	167 (15.0)	235 (16.8)	1,629 (17.2)
Female	596 (43.8)	637 (45.5)	651 (46.5)	643 (45.9)	655 (46.8)	488 (43.7)	646 (46.2)	4,316 (45.5)
**Clinical characteristics**								
Dysentery^*a*^	206 (15.1)	148 (10.6)	138 (9.9)	93 (6.6)	234 (16.7)	170 (15.2)	241 (17.2)	1,230 (13.0)
Dehydration^*b*^								
None	1,191 (87.5)	656 (46.9)	1,348 (96.4)	1,236 (88.3)	1,185 (84.6)	214 (19.2)	1,310 (93.6)	7,140 (75.3)
Some	168 (12.3)	685 (48.9)	49 (3.5)	154 (11.0)	207 (14.8)	897 (80.3)	70 (5.0)	2,230 (23.5)
Severe	2 (0.1)	59 (4.2)	2 (0.1)	10 (0.7)	8 (0.6)	6 (0.5)	19 (1.4)	106 (1.1)
Hospitalized	125 (9.2)	80 (5.7)	15 (1.1)	1 (0.1)	25 (1.8)	15 (1.3)	102 (7.3)	363 (3.8)
Received antibiotics prior to enrollment	237 (17.4)	33 (2.4)	54 (3.9)	53 (3.8)	77 (5.5)	60 (5.4)	42 (3.0)	556 (5.9)
**Rectal swab information** ^*c*^								
Cary-Blair	1,361 (100.0)	1,400 (100.0)	1,399 (100.0)	1,400 (100.0)	1,400 (100.0)	1,117 (100.0)	1,399 (100.0)	9,476 (100.0)
mBGS	1,361 (100.0)	1,400 (100.0)	1,399 (100.0)	1,400 (100.0)	1,400 (100.0)	1,117 (100.0)	1,399 (100.0)	9,476 (100.0)
Rectal swabs collected from whole stool sample instead^*d*^	0 (0.0)	0 (0.0)	3 (0.2)	2 (0.1)	51 (3.6)	2 (0.2)	8 (0.6)	66 (0.7)
Rectal swab available for accessioning^*e*^								
Cary-Bair	1,361 (100.0)	1,400 (100.0)	1,399 (100.0)	1,400 (100.0)	1,400 (100.0)	1,117 (100.0)	1,399 (100.0)	9,476 (100.0)
mBGS	1,361 (100.0)	1,400 (100.0)	1,399 (100.0)	1,400 (100.0)	1,400 (100.0)	1,117 (100.0)	1,399 (100.0)	9,476 (100.0)
Time from collection to placement in transport media (min)								
Cary-Blair	2 [1, 2]	1 [1, 2]	1 [1, 2]	1 [0, 1]	1 [1, 1]	2 [1, 2]	1 [0, 1]	1 [1, 2]
mBGS	1 [1, 2]	1 [1, 2]	1 [1, 1]	0 [0, 1]	1 [0, 1]	2 [1, 2]	1 [0, 1]	1 [1, 1]
Time from collection to arrival at central laboratory (hours)								
Cary-Blair	3.25 [2.33, 4.17]	3.88 [2.93, 4.82]	4.35 [3.67, 4.97]	4.08 [2.35, 5.63]	2.32 [1.75, 3.05]	2.58 [1.67, 3.37]	1.43 [0.83, 2.43]	3.13 [1.93, 4.30]
mBGS	3.25 [2.33, 4.17]	3.88 [2.93, 4.82]	4.33 [3.67, 4.97]	4.08 [2.36, 5.63]	2.32 [1.75, 3.04]	2.58 [1.67, 3.37]	1.43 [0.83, 2.45]	3.13 [1.95, 4.30]
Temperature excursion^*f*^								
None	1,355 (99.6)	1,314 (93.9)	1,396 (99.8)	1,343 (95.9)	1,268 (90.6)	1,071 (95.9)	1,372 (98.1)	9,119 (96.2)
Brief	6 (0.4)	86 (6.1)	3 (0.2)	24 (1.7)	103 (7.4)	7 (0.6)	20 (1.4)	249 (2.6)
Moderate	0 (0.0)	0 (0.0)	0 (0.0)	33 (2.4)	11 (0.8)	39 (3.5)	4 (0.3)	87 (0.9)
Prolonged	0 (0.0)	0 (0.0)	0 (0.0)	0 (0.0)	0 (0.0)	0 (0.0)	0 (0.0)	0 (0.0)
Unknown	0 (0.0)	0 (0.0)	0 (0.0)	0 (0.0)	18 (1.3)	0 (0.0)	3 (0.2)	21 (0.2)
**Whole stool information**								
Whole stool sample collected^*g*^								
Cary-Blair	669 (49.2)	0 (0.0)	0 (0.0)	0 (0.0)	0 (0.0)	0 (0.0)	463 (33.1)	1132 (11.9)
mBGS	428 (31.4)	0 (0.0)	0 (0.0)	0 (0.0)	0 (0.0)	0 (0.0)	512 (36.6)	940 (9.9)
Whole stool available for accessioning								
Cary-Blair	669 (100.0)	–^*h*^	–	–	–	–	463 (100.0)	1132 (100.0)
mBGS	428 (100.0)	–	–	–	–	–	512 (100.0)	940 (100.0)
Whole stool collected within 6 hours of rectal swab collection								
Cary-Blair	669 (100.0)	–	–	–	–	–	460 (99.4)	1129 (99.7)
mBGS	428 (100.0)	–	–	–	–	–	499 (97.5)	927 (98.6)
Time from collection to placement in transport media (min)								
Cary-Blair	2 [2, 2]	–	–	–	–	–	1 [0, 1]	2 [1, 2]
mBGS	2 [2, 2]	–	–	–	–	–	1 [1, 1]	2 [1, 2]
Time from collection to arrival at central laboratory (hours)								
Cary-Blair	3.25 [2.17, 4.17]	–	–	–	–	–	1.68 [0.80, 2.73]	2.55 [1.42, 3.83]
mBGS	2.68 [1.98, 3.58]	–	–	–	–	–	0.93 [0.53, 1.54]	1.63 [0.82, 2.83]
Temperature excursion^*f*^								
None	1,093 (99.6)	–	–	–	–	–	942 (98.2)	2,035 (99.0)
Brief	4 (0.4)	–	–	–	–	–	12 (1.3)	16 (0.8)
Moderate	0 (0.0)	–	–	–	–	–	2 (0.2)	2 (0.1)
Prolonged	0 (0.0)	–	–	–	–	–	0 (0.0)	0 (0.0)
Unknown	0 (0.0)	–	–	–	–	–	3 (0.3)	3 (0.1)

^*a*^ Blood in the stool as reported by caregiver during the diarrheal episode or by clinician diagnosis.

^*b*^ Based on ICMI criteria classification. Severe dehydration = At least two of the following signs: lethargy, abnormally sunken eyes, drinks poorly, skin pinch >2 seconds. Some dehydration = At least two of the following signs: restless/irritable, abnormally sunken eyes, drinks eagerly, skin pinch 1–2 s.

^*c*^ Two swabs are expected per child, one to be transported in Cary-Blair media and one to be transported in mBGS media.

^*d*^ Rectal swabs may be produced from a whole stool sample if unable to be collected directly from the child’s rectum.

^*e*^ Samples may be unable to be accepted by the laboratory if they arrive improperly sealed or improperly labeled.

^*f*^ Temperature during transport was tracked using a WarmMark monitor.

^*g*^ One whole stool sample is expected per child. The first half of the study used Cary-Blair media and the second half used mBGS.

^*h*^ “–” indicates not applicable.

There was no overall statistical evidence (*P* > 0.99) of a difference in *Shigella* culture isolation proportion between CB (7.8%) and mBGS (7.9%) (Table 2) except in Bangladesh, where the isolation proportion was higher in mBGS (12.9% vs. 14.3%, *P* = 0.008). There were also no major differences in *Shigella* isolation between the two transport media within *Shigella* serotypes. We also compared the isolation proportion of the two media separately in children with dysentery and acute watery diarrhea. No significant difference was observed (*P* = 0.576 and *P* = 0.773, respectively, Table S1). Despite there being no major differences in overall isolation proportions between the two media, the two swabs, each transported in unique media, did not always identify the same children as having *Shigella*. There were 131 (15.3%) cases of *Shigella* isolated from the CB swab and not the mBGS swab and 142 (16.6%) isolated from the mBGS swab and not the CB swab (out of the 856 *Shigella* culture confirmed cases identified in total, Table S2). Overall, this represents 97.0% agreement with a Cohen’s Kappa of 0.79 (Table S2). Irrespective of transport media, two swabs were superior at identifying children with *Shigella* compared with one: 747 (7.9%) of participants had *Shigella* isolated when considering only each child’s first swab, and 881 (9.3%) when both swabs were included (*P* < 0.001, Table 3).

**TABLE 2:** Comparison of *Shigella* culture positivity in Cary Blair and mBGS transport media stratified by site and serotype^*a*^

Country	N	*Shigella* culture positivity^*b*^: n (%)				Difference^*c*^ % (CI)	*P*-value^*c*^	
		Cary Blair		mBGS			Raw	Holm-adjusted^*d*^
		n	% (CI)	n	% (CI)			
All *Shigella* isolates								
Bangladesh	1,361	175	12.9 (11.2, 14.7)	195	14.3 (12.6, 16.3)	1.5 (0.7, 2.3)	0.001	0.008
Kenya	1,400	68	4.9 (3.8, 6.1)	67	4.8 (3.8, 6.0)	−0.1 (−0.8, 0.7)	>0.99	>0.99
Malawi	1,399	73	5.2 (4.2, 6.5)	78	5.6 (4.5, 6.9)	0.4 (−0.6, 1.3)	0.560	>0.99
Mali	1,400	69	4.9 (3.9, 6.2)	65	4.6 (3.7, 5.9)	−0.3 (−1.0, 0.5)	0.571	>0.99
Pakistan	1,400	131	9.4 (7.9, 11.0)	120	8.6 (7.2, 10.2)	−0.8 (−1.9, 0.4)	0.222	>0.99
Peru^*e*^	843	64	7.6 (6.0, 9.6)	65	7.7 (6.1, 9.7)	0.1 (−1.5, 1.7)	>0.99	>0.99
The Gambia	1,399	134	9.6 (8.1, 11.2)	135	9.6 (8.2, 11.3)	0.1 (−0.6, 0.7)	>0.99	>0.99
Overall	9,202	714	7.8 (7.2, 8.3)	725	7.9 (7.3, 8.4)	0.1 (−0.2, 0.5)	0.545	>0.99
*S. flexneri*								
Bangladesh	1,361	85	6.2 (5.1, 7.7)	93	6.8 (5.6, 8.3)	0.6 (0.1, 1.1)	0.043	0.344
Kenya	1,400	40	2.9 (2.1, 3.9)	37	2.6 (1.9, 3.6)	−0.2 (−0.8, 0.4)	0.646	>0.99
Malawi	1,399	25	1.8 (1.2, 2.6)	32	2.3 (1.6, 3.2)	0.5 (−0.1, 1.1)	0.169	>0.99
Mali	1,400	47	3.4 (2.5, 4.4)	44	3.1 (2.4, 4.2)	−0.2 (−0.8, 0.3)	0.606	>0.99
Pakistan	1,400	72	5.1 (4.1, 6.4)	62	4.4 (3.5, 5.6)	−0.7 (−1.5, 0.1)	0.123	0.861
Peru^*e*^	843	45	5.3 (4.0, 7.1)	46	5.5 (4.1, 7.2)	0.1 (−1.3, 1.5)	>0.99	>0.99
The Gambia	1,399	85	6.1 (4.9, 7.4)	88	6.3 (5.1, 7.7)	0.2 (−0.4, 0.9)	0.663	>0.99
Overall	9,202	399	4.3 (3.9, 4.8)	402	4.4 (4.0, 4.8)	0.0 (−0.2, 0.3)	0.872	>0.99
*S. sonnei*								
Bangladesh	1,361	57	4.2 (3.2, 5.4)	66	4.8 (3.8, 6.1)	0.7 (0.2, 1.1)	0.016	0.128
Kenya	1,400	20	1.4 (0.9, 2.2)	21	1.5 (1.0, 2.3)	0.1 (−0.3, 0.4)	>0.99	>0.99
Malawi	1,399	31	2.2 (1.6, 3.1)	31	2.2 (1.6, 3.1)	0.0 (−0.6, 0.6)	>0.99	>0.99
Mali	1,400	11	0.8 (0.4, 1.4)	11	0.8 (0.4, 1.4)	0.0 (−0.3, 0.3)	>0.99	>0.99
Pakistan	1,400	32	2.3 (1.6, 3.2)	32	2.3 (1.6, 3.2)	0.0 (−0.6, 0.6)	>0.99	>0.99
Peru^*e*^	843	19	2.2 (1.4, 3.5)	19	2.2 (1.4, 3.5)	0.0 (−0.9, 0.9)	>0.99	>0.99
The Gambia	1,399	38	2.7 (2.0, 3.7)	37	2.6 (1.9, 3.6)	−0.1 (−0.2, 0.1)	>0.99	>0.99
Overall	9,202	208	2.3 (2.0, 2.6)	217	2.4 (2.1, 2.7)	0.1 (−0.1, 0.3)	0.356	>0.99
*S. boydii*								
Bangladesh	1,361	24	1.8 (1.2, 2.6)	23	1.7 (1.1, 2.5)	−0.1 (−0.3, 0.2)	>0.99	>0.99
Kenya	1,400	5	0.4 (0.1, 0.8)	6	0.4 (0.2, 0.9)	0.1 (−0.1, 0.2)	>0.99	>0.99
Malawi	1,399	10	0.7 (0.4, 1.3)	7	0.5 (0.2, 1.0)	−0.2 (−0.6, 0.2)	0.450	>0.99
Mali	1,400	9	0.6 (0.3, 1.2)	9	0.6 (0.3, 1.2)	0.0 (−0.4, 0.4)	>0.99	>0.99
Pakistan	1,400	15	1.1 (0.6, 1.8)	15	1.1 (0.6, 1.8)	0.0 (−0.4, 0.4)	>0.99	>0.99
Peru^*e*^	843	0	0 (−)	0	0 (−)	–^*f*^	–	–
The Gambia	1,399	8	0.6 (0.3, 1.1)	7	0.5 (0.2, 1.0)	−0.1 (−0.2, 0.1)	>0.99	>0.99
Overall	9,202	71	0.8 (0.6, 1.0)	67	0.7 (0.6, 0.9)	−0.0 (−0.2, 0.1)	0.571	>0.99
*S. dysenteriae*								
Bangladesh	1,361	7	0.5 (0.2, 1.1)	10	0.7 (0.4, 1.4)	0.2 (−0.0, 0.5)	0.248	0.992
Kenya	1,400	0	0 (−)	2	0.1 (0.0, 0.5)	–	–	–
Malawi	1,399	7	0.5 (0.2, 1.0)	8	0.6 (0.3, 1.1)	0.1 (−0.2, 0.3)	>0.99	>0.99
Mali	1,400	2	0.1 (0.0, 0.5)	1	0.1 (0.0, 0.4)	−0.1 (−0.2, 0.1)	>0.99	>0.99
Pakistan	1,400	8	0.6 (0.3, 1.1)	9	0.6 (0.3, 1.2)	0.1 (−0.2, 0.3)	>0.99	>0.99
Peru^*e*^	843	0	0 (−)	0	0 (−)	–	–	–
The Gambia	1,399	1	0.1 (0.0, 0.4)	1	0.1 (0.0, 0.4)	0.0 (0.0, 0.0)	–	–
Overall	9,202	25	0.3 (0.2, 0.4)	31	0.3 (0.2, 0.5)	0.1 (−0.0, 0.1)	0.149	0.745

^*a*^ CI: 95% confidence interval, mBGS: modified buffered glycerol saline.

^*b*^ Culture *Shigella* culture positivity is defined as the percentage of enrolled participants with *Shigella* isolated from culture out of total participants with both rectal swabs cultured using both Cary-Blair and mBGS transport media.

^*c*^ McNemar’s test of superiority.

^*d*^ *P*-values were adjusted for multiple comparison testing within each species using the Holm-Bonferroni method to control familywise error rate. Adjusted *P*-values <0.05 are considered significant.

^*e*^ Includes only participants enrolled after January 19, 2023, due to a laboratory error that made previous results incomparable across media types.

^*f*^ “–” indicates not applicable because whole stool was not collected as part of whole stool substudy.

**TABLE 3:** Comparison of *Shigella* isolation comparing one swab sample to two swab samples

	Enrolled	*Shigella* culture positivity n (%)		Difference % (CI)	Raw *P*-value^*a*^	Holm-Adjusted *P*-value^*a*^^,*b*^
		First swab	Either swab			
All *Shigella* serotypes						
Bangladesh	1,361	180 (13.2%)	200 (14.7%)	−1.47 (−2.11, −0.83%)	<0.001	<0.001
Kenya	1,400	70 (5.0%)	82 (5.9%)	−0.86 (−1.34, −0.37%)	0.001	0.001
Malawi	1,399	76 (5.4%)	99 (7.1%)	−1.64 (−2.31, −0.98%)	<0.001	<0.001
Mali	1,400	70 (5.0%)	81 (5.8%)	−0.79 (−1.25, −0.32%)	0.001	0.001
Pakistan	1,400	126 (9.0%)	159 (11.4%)	−2.36 (−3.15, −1.56%)	<0.001	<0.001
Peru	1,117	93 (8.3%)	114 (10.2%)	−1.88 (−2.68, −1.08%)	<0.001	<0.001
The Gambia	1,399	132 (9.4%)	146 (10.4%)	−1.00 (−1.52, −0.48%)	<0.001	0.001
Overall	9,476	747 (7.9%)	881 (9.3%)	−1.41 (−1.65, −1.18%)	<0.001	<0.001
*S. flexneri*						
Bangladesh	1,361	86 (6.3%)	95 (7.0%)	−0.66 (−1.09, −0.23%)	0.003	0.008
Kenya	1,400	39 (2.8%)	48 (3.4%)	−0.64 (−1.06, −0.22%)	0.003	0.008
Malawi	1,399	26 (1.9%)	38 (2.7%)	−0.86 (−1.34, −0.37%)	0.001	0.002
Mali	1,400	47 (3.4%)	53 (3.8%)	−0.43 (−0.77, −0.09%)	0.014	0.014
Pakistan	1,400	67 (4.8%)	84 (6.0%)	−1.21 (−1.79, −0.64%)	<0.001	<0.001
Peru	1,117	65 (5.8%)	80 (7.2%)	−1.34 (−2.02, −0.67%)	<0.001	0.001
The Gambia	1,399	83 (5.9%)	97 (6.9%)	−1.00 (−1.52, −0.48%)	<0.001	0.001
Overall	9,476	413 (4.4%)	495 (5.2%)	−0.87 (−1.05, −0.68%)	<0.001	<0.001
*S. sonnei*						
Bangladesh	1,361	58 (4.3%)	67 (4.9%)	−0.66 (−1.09, −0.23%)	0.003	0.013
Kenya	1,400	23 (1.6%)	24 (1.7%)	−0.07 (−0.21, 0.07%)	0.317	0.317
Malawi	1,399	32 (2.3%)	40 (2.9%)	−0.57 (−0.97, −0.18%)	0.005	0.019
Mali	1,400	11 (0.8%)	13 (0.9%)	−0.14 (−0.34, 0.06%)	0.157	0.315
Pakistan	1,400	32 (2.3%)	42 (3.0%)	−0.71 (−1.16, −0.27%)	0.002	0.009
Peru	1,117	28 (2.5%)	33 (3.0%)	−0.45 (−0.84, −0.06%)	0.025	0.076
The Gambia	1,399	38 (2.7%)	38 (2.7%)	0.00 (0.00, 0.00%)	–^*c*^	–
Overall	9,476	222 (2.3%)	257 (2.7%)	−0.37 (−0.49, −0.25%)	<0.001	<0.001
*S. boydii*						
Bangladesh	1,361	25 (1.8%)	25 (1.8%)	0.00 (0.00, 0.00%)	–	–
Kenya	1,400	6 (0.4%)	6 (0.4%)	0.00 (0.00, 0.00%)	–	–
Malawi	1,399	10 (0.7%)	12 (0.9%)	−0.14 (−0.34, 0.06%)	0.157	0.315
Mali	1,400	10 (0.7%)	13 (0.9%)	−0.21 (−0.46, 0.03%)	0.083	0.250
Pakistan	1,400	17 (1.2%)	19 (1.4%)	−0.14 (−0.34, 0.06%)	0.157	0.315
Peru	1,117	0 (0.0%)	0 (0.0%)	0.00 (0.00, 0.00%)	–	–
The Gambia	1,399	8 (0.6%)	8 (0.6%)	0.00 (0.00, 0.00%)	–	–
Overall	9,476	76 (0.8%)	83 (0.9%)	−0.07 (−0.13, −0.02%)	0.008	0.033
*S. dysenteriae*						
Bangladesh	1,361	9 (0.7%)	10 (0.7%)	−0.07 (−0.22, 0.07%)	0.317	0.635
Kenya	1,400	0 (0.0%)	2 (0.1%)	−0.14 (−0.34, 0.06%)	–	–
Malawi	1,399	8 (0.6%)	9 (0.6%)	−0.07 (−0.21, 0.07%)	0.317	0.635
Mali	1,400	2 (0.1%)	2 (0.1%)	0.00 (0.00, 0.00%)	–	–
Pakistan	1,400	8 (0.6%)	10 (0.7%)	−0.14 (−0.34, 0.06%)	0.157	0.472
Peru	1,117	0 (0.0%)	1 (0.1%)	−0.09 (−0.26, 0.09%)	–	–
The Gambia	1,399	1 (0.1%)	1 (0.1%)	0.00 (0.00, 0.00%)	–	–
Overall	9,476	28 (0.3%)	35 (0.4%)	−0.07 (−0.13, −0.02%)	0.008	0.033

^*a*^ McNemar’s test.

^*b*^ *P*-values were adjusted for multiple comparison testing within each species using the Holm-Bonferroni method to control familywise error rate. Adjusted *P*-values <0.05 are considered significant.

^*c*^ “–” indicates not applicable.

Among the 2,048 children with both whole stool and rectal swab samples, *Shigella* was isolated from 235 (11.5%) participants from both whole stool and rectal swab samples, whereas 19 (0.09%) participants had *Shigella* isolated from rectal swab only and 25 (1.2%) from whole stool only (Table S3). Rectal swabs were no worse than whole stool at isolating *Shigella* by culture (12.4% and 12.7%, respectively) with a difference of −0.29% (95% CI: −0.83% to 0.24%; Table 4). In site and serotype-specific stratification, there was no evidence of non-inferiority in The Gambia, but in Bangladesh, we could not reject the null hypothesis that rectal swabs were non-inferior (13.40%) to whole stool (14.22%) (difference: −0.82% [−1.51%, −0.13%]) among all species (Table 4). However, rectal swabs were non-inferior to whole stool in Bangladesh when both rectal swabs were compared with whole stool instead of only one swab (14.68% isolation vs. 14.22% isolation, respectively; difference 0.46% [95%CI: 0.06%, 0.85%], Table S4) and remained non-inferior when subset by species. Including both of a child’s rectal swabs in the comparison to whole stool, 252 (12.3%) participants had *Shigella* isolated from both sample types, 26 (1.3%) were identified by rectal swab only, and 8 (0.04%) identified from whole stool only (Table S5).

**TABLE 4:** Country-specific and overall *Shigella* culture positivity from rectal swab and whole stool samples, across sites involved in the whole stool/rectal swab comparison sub-study matched by transport media^*a*^

Country	N	*Shigella* culture positivity^*b*^		
		Rectal swab: n (%)^*c*^	Whole stool: n (%)^*d*^	Difference: % (CI)
All *Shigella* isolates				
Bangladesh	1,097	147 (13.40)	156 (14.22)	−0.82 (−1.51, −0.13)
The Gambia	951	107 (11.25)	104 (10.94)	0.32 (−0.52, 1.15)
Overall	2,048	254 (12.40)	260 (12.70)	−0.29 (−0.83, 0.24)
*S. flexneri*				
Bangladesh	1,097	70 (6.38)	73 (6.65)	−0.27 (−0.72, 0.18)
The Gambia	951	70 (7.36)	66 (6.94)	0.42 (−0.32, 1.16)
Overall	2,048	140 (6.84)	139 (6.79)	0.05 (−0.37, 0.47)
*S. sonnei*				
Bangladesh	1,097	47 (4.28)	53 (4.83)	−0.55 (−1.02, −0.07)
The Gambia	951	29 (3.05)	29 (3.05)	0.00 (−0.25, 0.25)
Overall	2,048	76 (3.71)	82 (4.00)	−0.29 (−0.57, −0.01)
*S. boydii*				
Bangladesh	1,097	19 (1.73)	18 (1.64)	0.09 (−0.17, 0.35)
The Gambia	951	5 (0.53)	5 (0.53)	0.00 (−0.25, 0.25)
Overall	2,048	24 (1.17)	23 (1.12)	0.05 (−0.13, 0.23)
*S. dysenteriae*				
Bangladesh	1,097	9 (0.82)	10 (0.91)	−0.09 (−0.24, 0.06)
The Gambia	951	1 (0.11)	2 (0.21)	−0.11 (−0.28, 0.07)
Overall	2,048	10 (0.49)	12 (0.59)	−0.10 (−0.21, 0.02)

^*a*^ CI: 90% confidence interval, mBGS: modified-buffered glycerol saline, Prop: proportion.

^*b*^ *Shigella* culture positivity is defined as the percentage of participants with *Shigella* isolated from culture out of total participants with both a rectal swab and a whole stool sample collected for the *Shigella* culture comparison.

^*c*^ Includes only the first collected rectal swab from each child.

^*d*^ Whole stool was collected among children who produce a sample while still at the enrollment facility.

**Fig 1:**
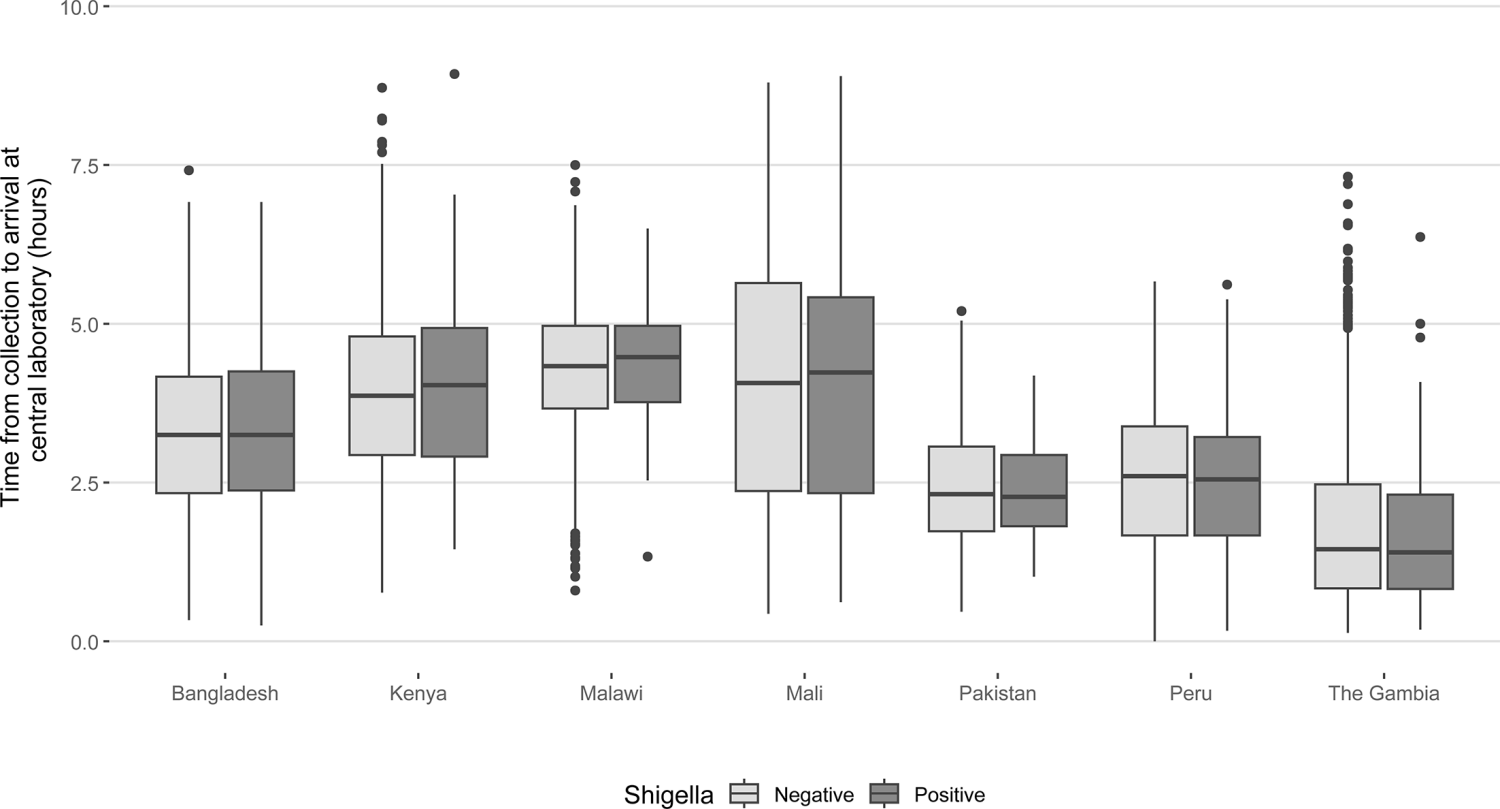
Time from sample collection to arrival at central laboratory by *Shigella* culture positivity.

**Fig 2:**
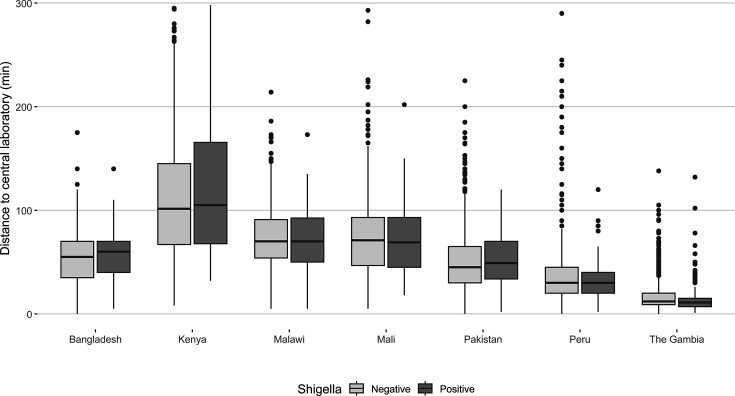
Comparison of *Shigella* culture positivity by travel distance (in min) from the clinic to central laboratory.

No meaningful differences were observed in *Shigella* isolation proportions from rectal swabs by timing between collection and placement in transport media, between collection and arrival at the central laboratory for primary plating, and by whether or not appropriate cold chain was maintained during specimen shipment (Table 5; Fig. 1 and 2).

**TABLE 5:** Comparison of the time (in min) of stool processing procedures by *Shigella* culture positivity by site and *Shigella* spp.^*a*^

	Bangladesh		Kenya		Malawi		Mali		Pakistan		Peru		The Gambia	
*Shigella*	−	+	−	+	−	+	−	+	−	+	−	+	−	+
N	1,166	195	1,333	67	1,321	78	1,335	65	1,280	120	1,044	73	1,264	135
Swab collection to placement in transport media (mins)	1.5 (1.5, 1.5)	1.5 (1.5, 1.5)	1.5 (1.0, 1.5)	1.5 (1.0, 1.5)	1.0 (0.5, 1.5)	1.0 (0.625, 1.5)	0.5 (0.5, 0.5)	0.5 (0.5, 0.5)	1.0 (0.5, 1.5)	1.0 (0.5, 1.5)	1.5 (1.5, 1.5)	1.5 (1.5, 1.5)	0.5 (0.0, 1.0)	0.5 (0.5, 1.0)
Transport media placement to placement in cool box (mins)	1.5 (1.5, 1.5)	1.5 (1.5, 1.5)	1.5 (1.5, 1.5)	1.5 (1.5, 1.5)	170.5 (140, 203.5)	175.25 (143.5, 217.3)	0.5 (0.5, 0.5)	0.5 (0.5, 0.5)	1.5 (1.0, 2.5)	2.0 (1.0, 2.5)	3.5 (2.5, 4.5)	3.5 (2.0, 4.0)	1.0 (0.5, 1.5)	1.0 (0.5, 1.5)
Placement in cool box to departure from clinic (mins)	137 (77, 187)	133 (77, 194.5)	106 (52, 172)	97 (39, 163.5)	5.0 (4, 10)	6.0 (4, 10)	161 (76, 255)	178 (76, 254)	85 (48, 128)	84 (53.75, 117)	112 (54.75, 160)	122 (62, 153)	68.5 (34, 128)	68 (36, 115.5)
Departure from clinic to arrival at central lab for inoculation (mins)	55 (35, 70)	60 (40, 70)	102 (67, 146)	105 (68.5, 168)	70 (54, 91)	70 (50, 92.5)	71 (46, 93)	69 (45, 93)	45 (30, 65)	49 (33.75, 70)	30 (20, 45)	30 (15.0, 39)	12 (9.0, 20)	11 (7, 15)

^*a*^ Median and interquartile range (IQR).

## DISCUSSION

*Shigella* is challenging to detect by microbiologic culture; therefore, optimizing conditions and sample types are critical to ensuring appropriate classification of *Shigella*-confirmed diarrhea. In this multi-country *Shigella*-focused study, we found that two transport media, CB and mBGS, yield similar *Shigella* isolation proportions; that a single rectal swab is sufficient to isolate *Shigella* compared with whole stool, and that two swabs yield optimal *Shigella* isolation proportions. We also found that there may be some site-specific nuances to optimal methods and likely unique practicalities that will be important considerations for eventual vaccine trials.

CB medium is the Center for Disease Control-recommended transport media for rectal swabs, known for its optimal buffering capacity and nutrient composition, which supports enteric pathogen preservation (24). Pre-packaged flocked rectal swabs in CB medium have become commercially available, which significantly improves reproducibility, scalability, and reduces preparation time. However, these swabs are more expensive and can have a relatively short shelf-life. We found CB was not superior to mBGS and, in fact, found mBGS to be superior to CB in isolating *Shigella* in Bangladesh, which has also been shown previously (20). Although CB mainly contains sodium thioglycolate, sodium chloride, and mono- and di-potassium phosphate, mBGS contains glycerol and saline, which together help maintain the stability of bacteria during transportation and provide better conservation of *Shigella* (20). However, unlike CB, mBGS requires preparation and assembly, which brings practical challenges. Although the individual reagents of mBGS media are available, rectal swabs in mBGS are not commercially available and must be prepared prior to use. Although mBGS is cost-effective, these extra steps require additional coordination and standardization, which may make it less practical for large-scale field studies.

*Shigella* culture positivity proportion from rectal swabs and whole stool samples collected across The Gambia and Bangladesh was highly comparable. This has also been demonstrated by previous studies (13, 15, 16), studies that have also highlighted the practical advantage of rectal swabs enabling quick sample collection rather than waiting for whole stool to be produced. Immediate sample collection is particularly advantageous for a bacterium such as *Shigella,* which is likely to be impacted by antibiotic use, which might be provided soon after presentation to a health facility (15). The current study aimed to collect specimens preferably before antibiotic use; however, pre-treatment occurred at several sites, reflecting local healthcare-seeking patterns. Given *Shigella’s* susceptibility to antibiotics, prior exposure could have inhibited bacterial growth; however, we expect pre-sample collection antibiotic use to be equally distributed across the comparison groups. At the Bangladesh site only, whole stool yielded slightly higher *Shigella* culture positivity; however, this difference went away when two rectal swabs were used instead of one.

Isolation proportions are impacted by factors such as transport media type, shipment temperature, and time between collection and initial processing (20, 24–26). These factors must be carefully balanced to preserve pathogen survival while considering cost-effectiveness and logistical challenges. Because EFGH followed strict protocols and QA/QC to ensure 2°C–8°C temperatures were maintained during shipment and that inoculation on the primary plate occurred less than 16 h from sample collection, we had little variability in these circumstances to explore their impact on *Shigella* isolation. In addition, although standard operating procedures were followed across all sites, subtle differences in staff competency, processing time, and environmental conditions could also have introduced bias. Given that refrigeration is known to increase the yield of enteric bacteria, our results may not be generalizable to settings without refrigeration. Our methodology was based on previous evaluation studies that showed that the recovery of the enteric pathogens in stool specimens held in Cary-Blair or BGS at refrigeration temperatures was much higher than the yield from the same specimens held at room temperature under the same environmental conditions (21, 27). Findings from this study can only be generalized to highly controlled research studies and not real-world settings where such standardized protocols and supplies as well as refrigeration are less controlled.

This study specifically focused on isolating *Shigella* to inform future vaccine trial protocols, which will include standardized protocols, strict oversight, and likely centralized supply procurement. However, future studies are necessary to evaluate the utility of these sample types and transport media for recovering multiple diarrheal bacterial pathogens and for recovering these pathogens in real-world settings, such as those without refrigeration.

The EFGH study demonstrated high culture isolation proportions of *Shigella* by thorough attention to proper sample collection, transit, and media. To maximize *Shigella* recovery by culture, while weighing practical considerations for eventual multi-country vaccine trials where standardization and minimizing missed *Shigella* cases will be critical, we recommend collection of two flocked rectal swabs, transported in CB or mBGS media, and strict adherence to optimal transit temperatures and times, all of which was feasible at EFGH sites.

## Data Availability

The EFGH statistical analysis plan (https://clinicaltrials.gov/study/NCT06047821) and study protocol (https://academic.oup.com/ofid/issue/11/Supplement_1) were made publicly available. The data sets were deidentified and are publicly available on Vivli (https://search.vivli.org/doiLanding/studies/PR00011860).

## SUPPLEMENTAL MATERIAL

Supplemental tables jcm.01279-25-s0001.docx Tables S1 to S5.

ASM does not own the copyrights to Supplemental Material that may be linked to, or accessed through, an article. The authors have granted ASM a non-exclusive, world-wide license to publish the Supplemental Material files. Please contact the corresponding author directly for reuse.
