# Supplementary material for: Optimizing Shigella isolation: a multi-site evaluation of laboratory culture methods for Shigella detection, speciation, and serotyping with different transport media and sample types in the Enterics for Global Health study
Source: J Clin Microbiol. 2026 Feb 26;64(4):e01279-25. doi: 10.1128/jcm.01279-25 (PMC13059758; doi:10.1128/jcm.01279-25)
Supplement: Supplemental tables — Tables S1 to S5. [file jcm.01279-25-s0001.docx]

# **SUPPLEMENTARY MATERIALS**

## **Supplemental Table 1:** Comparison of *Shigella* culture positivity in Cary Blair and mBGS transport media stratified by dysentery and acute watery diarrhea

| **Country** |  | ***Shigella* culture positivity^1,2^: n (%)** | | | | **Difference^3^**  **% (CI)** |  |
| --- | --- | --- | --- | --- | --- | --- | --- |
|  |  | **Cary Blair** | | **mBGS** | |  | **p-value^3^** |
|  | **N** | **n** | **% (CI)** | **n** | **% (CI)** |  |  |
| **All *Shigella* isolates** |  |  |  |  |  |  |  |
| Watery Diarrhea | 8007 | 438 | 5.5 (5.0, 6.0) | 443 | 5.5 (5.0, 6.1) | 0.1 (-0.3, 0.4) | 0.773 |
| Dysentery^4^ | 1195 | 276 | 23.1 (20.8, 25.6) | 282 | 23.6 (21.3, 26.1) | 0.5 (-1.0, 2.0) | 0.576 |
| ***S. flexneri*** |  |  |  |  |  |  |  |
| Watery Diarrhea | 8007 | 207 | 2.6 (2.3, 3.0) | 207 | 2.6 (2.3, 3.0) | 0.0 (-0.2, 0.2) | >0.99 |
| Dysentery^4^ | 1195 | 192 | 16.1 (14.1, 18.3) | 195 | 16.3 (14.3, 18.5) | 0.3 (-1.0, 1.5) | 0.787 |
| ***S. sonnei*** |  |  |  |  |  |  |  |
| Watery Diarrhea | 8007 | 158 | 2.0 (1.7, 2.3) | 162 | 2.0 (1.7, 2.4) | 0.0 (-0.1, 0.2) | 0.694 |
| Dysentery^4^ | 1195 | 50 | 4.2 (3.2, 5.5) | 55 | 4.6 (3.5, 5.9) | 0.4 (-0.3, 1.1) | 0.332 |
| CI: 95% confidence interval, mBGS: modified buffered glycerol saline.  ^1^ Culture *Shigella* culture positivity is defined as the percentage of enrolled participants with *Shigella* isolated from culture out of total participants with both rectal swabs cultured using both Cary-Blair and mBGS transport media.  ^2^ Participants from the Peru site were excluded if enrolled before January 19, 2023 due to a laboratory error that made previous results incomparable across media types.  ^3^ McNemar’s test of superiority  ^4^ Blood in the stool as reported by caregiver during the diarrheal episode or by clinician diagnosis. | | | | | | | |

## **Supplemental** **Table 2:** Concordance in *Shigella* culture positivity by media type (Cary-Blair vs. mBGS)

## Concordance in *Shigella* culture positivity by sample type (rectal swab vs. whole stool)

|  | **Overall** | | | | Percent Agreement: 97.0% | | |
| --- | --- | --- | --- | --- | --- | --- | --- |
|  |  |  |  |  | Cohen’s Kappa: 0.79 | | |
|  |  |  | Cary-Blair | | | |  |
|  |  |  | No *Shigella* | | | *Shigella* | Total |
|  | mBGS | No *Shigella* | 8346 | | | 131 | 8477 |
|  |  | *Shigella* | 142 | | | 583 | 725 |
|  |  | Total | 8488 | | | 714 | 9202 |
|  | **Bangladesh** | | | | Percent Agreement: 97.8% | | |
|  |  |  |  |  | Cohen’s Kappa: 0.91 | | |
|  |  |  | Cary-Blair | | | |  |
|  |  |  | No *Shigella* | | | *Shigella* | Total |
|  | mBGS | No *Shigella* | 1161 | | | 5 | 1166 |
|  |  | *Shigella* | 25 | | | 170 | 195 |
|  |  | Total | 1186 | | | 175 | 1361 |
|  | **Kenya** | | | | Percent Agreement: 97.9% | | |
|  |  |  |  |  | Cohen’s Kappa: 0.77 | | |
|  |  |  | Cary-Blair | | | |  |
|  |  |  | No *Shigella* | | | *Shigella* | Total |
|  | mBGS | No *Shigella* | 1318 | | | 15 | 1333 |
|  |  | *Shigella* | 14 | | | 53 | 67 |
|  |  | Total | 1332 | | | 68 | 1400 |
|  | **Malawi** | | | | | Percent Agreement: 96.6% | |
|  |  |  |  |  |  | Cohen’s Kappa: 0.67 | |
|  |  |  | Cary-Blair | | | |  |
|  |  |  | No *Shigella* | | | *Shigella* | Total |
|  | mBGS | No *Shigella* | 1300 | | | 21 | 1321 |
|  |  | *Shigella* | 26 | | | 52 | 78 |
|  |  | Total | 1326 | | | 73 | 1399 |
|  | **Mali** | | | Percent Agreement: 98.0% | | | |
|  |  |  |  | Cohen’s Kappa: 0.78 | | | |
|  |  |  | Cary-Blair | | | |  |
|  |  |  | No *Shigella* | | | *Shigella* | Total |
|  | mBGS | No *Shigella* | 1319 | | | 16 | 1335 |
|  |  | *Shigella* | 12 | | | 53 | 65 |
|  |  | Total | 1331 | | | 69 | 1400 |
|  | **Pakistan** | | | Percent Agreement: 95.2% | | | |
|  |  |  |  | Cohen’s Kappa: 0.71 | | | |
|  |  |  | Cary-Blair | | | |  |
|  |  |  | No *Shigella* | | | *Shigella* | Total |
|  | mBGS | No *Shigella* | 1241 | | | 39 | 1280 |
|  |  | *Shigella* | 28 | | | 92 | 120 |
|  |  | Total | 1269 | | | 131 | 1400 |
|  | **Peru** | | | Percent Agreement: 94.2% | | | |
|  |  |  |  | Cohen’s Kappa: 0.59 | | | |
|  |  |  | Cary-Blair | | | |  |
|  |  |  | No *Shigella* | | | *Shigella* | Total |
|  | mBGS | No *Shigella* | 754 | | | 24 | 778 |
|  |  | *Shigella* | 25 | | | 40 | 65 |
|  |  | Total | 779 | | | 64 | 843 |
|  | **The Gambia** | | | Percent Agreement: 98.4% | | | |
|  |  |  |  | Cohen’s Kappa: 0.91 | | | |
|  |  |  | Cary-Blair | | | |  |
|  |  |  | No *Shigella* | | | *Shigella* | Total |
|  | mBGS | No *Shigella* | 1253 | | | 11 | 1264 |
|  |  | *Shigella* | 12 | | | 123 | 135 |
|  |  | Total | 1265 | | | 134 | 1399 |

## **Supplemental Table 3:** Two-by-two tables comparing *Shigella* positivity between rectal swab and whole stool using the first collected rectal swab matched with whole stool by media type.

|  | **Overall** | | | Percent Agreement: 97.9% | | |
| --- | --- | --- | --- | --- | --- | --- |
|  |  |  |  | Cohen’s Kappa: 0.90 | | |
|  |  |  | Whole Stool | | |  |
|  |  |  | No *Shigella* | | *Shigella* | Total |
|  | Rectal Swab | No *Shigella* | 1769 | | 25 | 1794 |
|  |  | *Shigella* | 19 | | 235 | 254 |
|  |  | Total | 1788 | | 260 | 2048 |
|  | **Bangladesh** | | | Percent Agreement: 98.1% | | |
|  |  |  |  | Cohen’s Kappa: 0.92 | | |
|  |  |  | Whole Stool | | |  |
|  |  |  | No *Shigella* | | *Shigella* | Total |
|  | Rectal Swab | No *Shigella* | 935 | | 15 | 950 |
|  |  | *Shigella* | 6 | | 141 | 147 |
|  |  | Total | 941 | | 156 | 1097 |
|  | **The Gambia** | | | Percent Agreement: 97.6% | | |
|  |  |  |  | Cohen’s Kappa: 0.88 | | |
|  |  |  | Whole Stool | | |  |
|  |  |  | No *Shigella* | | *Shigella* | Total |
|  | Rectal Swab | No *Shigella* | 834 | | 10 | 844 |
|  |  | *Shigella* | 13 | | 94 | 107 |
|  |  | Total | 847 | | 104 | 951 |

## **Supplemental Table 4:** Country-specific and overall *Shigella* culture positivity from rectal swab and whole stool samples, across sites involved in the whole stool/rectal swab comparison sub-study, including *Shigella* positivity in either rectal swab.

| **Country** |  | ***Shigella* culture positivity^1^** | | |
| --- | --- | --- | --- | --- |
|  | **N** | **Rectal swab: n (%)^2^** | **Whole stool: n (%)^3^** | **Difference: % (CI)** |
| **All *Shigella* isolates** |  |  |  |  |
| Bangladesh | 1097 | 161 (14.68) | 156 (14.22) | 0.46 (0.06, 0.85) |
| The Gambia | 951 | 117 (12.30) | 104 (10.94) | 1.37 (0.47, 2.27) |
| **Overall** | **2048** | **278 (13.57)** | **260 (12.70)** | **0.88 (0.41, 1.35)** |
| ***S. flexneri*** |  |  |  |  |
| Bangladesh | 1097 | 76 (6.93) | 73 (6.65) | 0.27 (0.01, 0.53) |
| The Gambia | 951 | 80 (8.41) | 66 (6.94) | 1.47 (0.66, 2.28) |
| Overall | 2048 | 156 (7.62) | 139 (6.79) | 0.83 (0.43, 1.23) |
| ***S. sonnei*** |  |  |  |  |
| Bangladesh | 1097 | 54 (4.92) | 53 (4.83) | 0.09 (-0.17, 0.35) |
| The Gambia | 951 | 29 (3.05) | 29 (3.05) | 0.00 (-0.25, 0.25) |
| Overall | 2048 | 83 (4.05) | 82 (4.00) | 0.05 (-0.13, 0.23) |
| ***S. boydii*** |  |  |  |  |
| Bangladesh | 1097 | 19 (1.73) | 18 (1.64) | 0.09 (-0.17, 0.35) |
| The Gambia | 951 | 5 (0.53) | 5 (0.53) | 0.00 (-0.25, 0.25) |
| Overall | 2048 | 24 (1.17) | 23 (1.12) | 0.05 (-0.13, 0.23) |
| ***S. dysenteriae*** |  |  |  |  |
| Bangladesh | 1097 | 10 (0.91) | 10 (0.91) | 0.00 (0.00, 0.00) |
| The Gambia | 951 | 1 (0.11) | 2 (0.21) | -0.11 (-0.28, 0.07) |
| Overall | 2048 | 11 (0.54) | 12 (0.59) | -0.05 (-0.13, 0.03) |
| CI: 90% confidence interval, mBGS: modified buffered glycerol saline, Prop: proportion. | | | | |
| ^1^ *Shigella* culture positivity is defined as the percentage of participants with *Shigella* isolated from culture out of total participants with both rectal swab and whole stool collected for the *Shigella* culture comparison.  ^2^ Includes *Shigella* positivity in either of two rectal swabs from each child. | | | | |
| ^3^ Whole stool was collected among children who produce a sample while still at the enrollment facility. | | | | |

## **Supplemental Table 5:** Two-by-two tables by site and *Shigella* species to compare *Shigella* positivity between rectal swab and whole stool using Shigella positivity of either rectal swab (matching the media type used for the same participant’s whole stool sample).

|  | **Overall** | | | Percent Agreement: 98.3% | | |
| --- | --- | --- | --- | --- | --- | --- |
|  |  |  |  | Cohen’s Kappa: 0.93 | | |
|  |  |  | Whole Stool | | |  |
|  |  |  | No *Shigella* | | *Shigella* | Total |
|  | Rectal Swab | No *Shigella* | 1762 | | 8 | 177 |
|  |  | *Shigella* | 26 | | 252 | 278 |
|  |  | Total | 1788 | | 260 | 2048 |
|  | **Bangladesh** | | | Percent Agreement: 99.4% | | |
|  |  |  |  | Cohen’s Kappa: 0.97 | | |
|  |  |  | Whole Stool | | |  |
|  |  |  | No *Shigella* | | *Shigella* | Total |
|  | Rectal Swab | No *Shigella* | 935 | | 1 | 936 |
|  |  | *Shigella* | 6 | | 155 | 161 |
|  |  | Total | 941 | | 156 | 1097 |
|  | **The Gambia** | | | Percent Agreement: 97.2% | | |
|  |  |  |  | Cohen’s Kappa: 0.87 | | |
|  |  |  | Whole Stool | | |  |
|  |  |  | No *Shigella* | | *Shigella* | Total |
|  | Rectal Swab | No *Shigella* | 827 | | 7 | 834 |
|  |  | *Shigella* | 20 | | 97 | 117 |
|  |  | Total | 847 | | 104 | 951 |
